# Seasonality modeling of the distribution of *Aedes albopictus* in China based on climatic and environmental suitability

**DOI:** 10.1186/s40249-019-0612-y

**Published:** 2019-12-03

**Authors:** Xueli Zheng, Daibin Zhong, Yulan He, Guofa Zhou

**Affiliations:** 1 Department of Pathogen Biology, School of Public Health, Southern Medical University, 1838 Guangzhou North Avenue, Guangzhou, 510515 China; 2 Program in Public Health, University of California, Irvine, CA USA

**Keywords:** *Aedes albopictus*, Distribution, Northern margin, China, Climate, Environment

## Abstract

**Background:**

*Aedes albopictus* is a highly invasive mosquito species and a major vector of numerous viral pathogens. Many recent dengue fever outbreaks in China have been caused solely by the vector. Mapping of the potential distribution ranges of *Ae. albopictus* is crucial for epidemic preparedness and the monitoring of vector populations for disease control. Climate is a key factor influencing the distribution of the species. Despite field studies indicating seasonal population variations, very little modeling work has been done to analyze how environmental conditions influence the seasonality of *Ae. albopictus.* The aim of the present study was to develop a model based on available observations, climatic and environmental data, and machine learning methods for the prediction of the potential seasonal ranges of *Ae. albopictus* in China.

**Methods:**

We collected comprehensive up-to-date surveillance data in China, particularly records from the northern distribution margin of *Ae. albopictus*. All records were assigned long-term (1970–2000) climatic data averages based on the WorldClim 2.0 data set. Machine learning regression tree models were developed using a 10-fold cross-validation method to predict the potential seasonal (or monthly) distribution ranges of *Ae. albopictus* in China at high resolution based on environmental conditions. The models were assessed based on sensitivity, specificity, and accuracy, using area under curve (AUC). WorldClim 2.0 and climatic and environmental data were used to produce environmental conduciveness (probability) prediction surfaces. Predicted probabilities were generated based on the averages of the 10 models.

**Results:**

During 1998–2017, *Ae. albopictus* was observed at 200 out of the 242 localities surveyed. In addition, at least 15 new *Ae. albopictus* occurrence sites lay outside the potential ranges that have been predicted using models previously. The average accuracy was 98.4% (97.1–99.5%), and the average AUC was 99.1% (95.6–99.9%). The predicted *Ae. albopictus* distribution in winter (December–February) was limited to a small subtropical-tropical area of China, and *Ae. albopictus* was predicted to occur in northern China only during the short summer season (usually June–September). The predicted distribution areas in summer could reach northeastern China bordering Russia and the eastern part of the Qinghai-Tibet Plateau in southwestern China. *Ae. albopictus* could remain active in expansive areas from central to southern China in October and November.

**Conclusions:**

Climate and environmental conditions are key factors influencing the seasonal distribution of *Ae. albopictus* in China. The areas predicted to potentially host *Ae. albopictus* seasonally in the present study could reach northeastern China and the eastern slope of the Qinghai-Tibet Plateau. Our results present new evidence and suggest the expansion of systematic vector population monitoring activities and regular re-assessment of epidemic risk potential.

## Multilingual abstracts

Please see Additional file 1 for translations of the abstract into the five official working languages of the United Nations.

## Background

*Aedes albopictus* (*Stegomyia albopicta*) Skuse, a mosquito native to the tropical and subtropical areas of south and east Asia, is an epidemiologically important vector of numerous viral pathogens, including yellow, dengue, chikungunya, and potentially Zika fever viruses [1–4]. An aggressive invasive species, *Ae. albopictus* has invaded and adapted to diverse environments in numerous countries across all continents except Antarctica [5]. The frequent outbreaks of dengue and chikungunya fevers in southern and southeastern China over the past few decades, and in central and eastern China in the past few years have raised major public health concerns [6–8]. In numerous cases, *Ae. albopictus* has been the sole vector responsible for dengue fever outbreaks in China [8, 9]. At present, vaccines are available for some *Aedes* mosquito transmitted diseases such as yellow fever and dengue fever. However, Sanofi Pasteur, the dengue fever vaccine manufacturer, announced in 2017 that people who receive the vaccine and have not been infected previously with a dengue virus may be at risk of developing severe dengue fever if they contract dengue after being vaccinated (https://www.cdc.gov/dengue/prevention/dengue-vaccine.html).

In addition, no therapeutic treatments are available for most viruses transmitted by *Ae. albopictus*, which makes vector control a key strategy for controlling the transmission of such diseases [5]. Therefore, understanding the biology, distribution, and factors influencing the expansion of its range could facilitate the formulation of effective vector control strategies.

Local environmental conditions are key factors influencing the distribution, survivorship, and development of both larval and adult *Ae. albopictus* mosquitoes [10, 11]. The high capacity of *Ae. albopictus* to adapt to diverse environments and climates makes it a potential invasive species in numerous localities globally [1–5, 12–15]. The dynamic expansion in range raises questions about how environmental factors influence the distribution of *Ae. albopictus*. For example, can *Ae. albopictus* extend its distribution range to temperate regions such as northern China? If the answer is ‘Yes’, since winters are very cold in northern China, can *Ae. albopictus* adults survive the winters or only emerge in summer in northern China?

Numerous studies have mapped regional or global distribution ranges of *Ae. albopictus* based on the biological or physiological characteristics of the species. Majority of the studies have examined how climatic conditions limit the distribution of the species, often focusing on temperature exclusively. Some studies have used laboratory-based results to predict how climatic factors, i.e., temperature and precipitation, affect the northern limits of *Ae. albopictus* in different regions [16–19] and potential ranges of *Ae. aegypti* and *Ae. albopictus* under present-day and future climate conditions [20]. Such studies present the most recent trends in research on *Aedes* risks. It is essential to examine how local environments influence *Ae. albopictus* ranges, particularly their seasonality in China, based on a more comprehensive review of field observational data since it could provide insights into the underlying factors [9, 13]. Prediction of seasonality is particularly critical because could facilitate better allocation of resources and guarantee cost-effectiveness in disease prevention and control.

In China, which has a vast territory, most regions are located in the temperate zone while some southern regions are located in the subtropical and tropical zones [21]. The northern region is close to the frigid zone. The Qinghai-Tibetan Plateau in southwest China is an area experiencing low temperatures throughout the year. There are also arid and semi-arid climates in the northwestern desert regions [21]. Notably, previous climatic suitability studies suggested that *Ae. albopictus* would be found only in the tropical, sub-tropical and warm temperate areas of China [20–22]. In a systematic surveillance that covered 19 provinces from 2006 to 2013, *Ae. albopictus* individuals were trapped in 16 out of the 19 provinces [23], which was consistent with previous reports [20, 22]. However, a more comprehensive review with updated information is required to evaluate the current distribution limits of *Ae. albopictus* in China.

Field studies from different areas have delineated high seasonality in *Ae. albopictus* population dynamics and distribution. For example, in Croatia, *Ae. albopictus* oviposition activity began in April and ended in November, with high seasonality and spatial heterogeneity [24]. In the northern temperate areas of Japan, adult *Ae. albopictus* were observed from May through October [25–28]. Similarly, in southern China, *Ae. albopictus* were active all year round [29]; however, in central China, adult *Ae. albopictus* were observed only from May to October [30]. Despite such findings in the field, modeling studies have rarely analyzed the seasonal influence of environment on *Ae. albopictus* populations and their distribution [18, 22, 31, 32].

In the present study, our aim was to model the seasonal distribution of *Ae. albopictus* in China based on comprehensive data from published literature. Our models used environmental data to predict distribution and seasonality, particularly in the earliest and latest months of occurrence of the species, at high spatial resolution. The present study focuses solely on the distribution of adult *Ae. albopictus.*

## Methods

Comprehensive *Ae. albopictus* field surveillance records and GPS location data from China for the 1998–2017 period were collected (Additional file 2: Table S1 and Additional file 3: Table S2). Since *Ae. albopictus* has been observed frequently in southern China [7–10], to minimize redundancy during modeling, repeated sampling activities in similar locations (e.g., the same city) in southern China (e.g., Guangdong, Zhejiang, and other provinces) were not included [7–10]. Information extracted from the surveillance records included prevalence and months of occurrence of *Ae. albopictus*. Incomplete occurrence period records were filled in using k-medoids clustering based on climate similarity (see Data analysis below). If a specific location was included in the published record, the GPS readings of that record were extracted. If only the name of the sampling location was included (usually the name of the city or county where the sample was collected), a GPS location was arbitrarily selected within the city or the major town (usually the capital) of the county. The GPS readings were used to extract the climatic and environmental data described in the following section.

### Climatic and environmental data for model development

We used WorldClim 2.0 as major reference climatic data [33]. High resolution (30 arcsec or approximately 1-km^2^ spatial resolution) WorldClim 2.0 data were generated based on averages during 1971–2000 [33]. Climatic data variables included monthly mean, minimum, and maximum temperatures, and monthly total precipitations. Climatic data that assigned to each sampling point were from the nearest grid points in WorldClim 2.0. Climatic-environmental zones were established based on previous literature [21]. The environmental regions were divided into four categories, including humid, sub-humid, semiarid, and arid regions. Climatic zone comprised nine categories, including south subtropical, mid-subtropical, north subtropical, warm temperate, mild temperate, cool temperate, plateau subtropical, plateau temperate, and plateau sub frigid zones. Each sampling location was assigned its corresponding climatic and environmental categories.

### Climatic data for spatial prediction

To map the spatial distribution of *Ae. albopictus* based on climatic-environmental factors in China, we used existing globally gridded climate surfaces WorldClim 2.0 data in the present study [33]. The WorldClim dataset has used extensively for the modeling of the impact of global climate change on the distribution of *Ae. albopictus*, particularly its range expansion, as well as the mapping of dengue and chikungunya fever risks in the US, Europe, and globally [13, 19, 31, 34]. WorldClim2.0 data includes globally gridded climate surfaces (30 arcsec or approximately 1-km^2^ spatial resolution) of monthly average temperature (minimum, mean, and maximum) and monthly rainfall for 1971–2000.

### Data analysis

Data analysis included three steps. Step 1: assigning climatic and environmental data to each surveillance record based on the GPS location of the record, regardless of the status of occurrence of *Ae. albopictus.* Step 2: sorting sampling points into groups based on similarity in environmental conditions and the known *Ae. albopictus* occurrence months, then assigning occurrence months to missing records due to lack of longitudinal observations, which was carried out using k-medoids clustering [35]. Step 3: predicting the conduciveness of environmental conditions for *Ae. albopictus* populations using a machine-learning regression model. The model was evaluated by examining *Ae. albopictus* occurrence against model-predicted probability of occurrence at each sampling site in each month. The cutoff predicted probability for determining ‘presence’ or ‘absence’ was based on accuracy [36], which balances specificity and sensitivity. The accuracy is calculated as the sum of correctly predicted positives and correctly predicted negatives over a total number of samples. The eventual model sensitivity and specificity were calculated based on a cutoff predicted probability that gives the optimal accuracy, i.e., if the predicted risk rate was greater than cutoff, the prediction was considered ‘presence’; otherwise it was considered ‘absence’. Sensitivity was estimated based on the proportion of sites with *Ae. albopictus* that were predicted as ‘presence’ and specificity was estimated based on the proportion of sites without *Ae. albopictus* that were predicted as ‘absence’. In addition to sensitivity, specificity, and accuracy, we also calculated area under curve (AUC) based on receiver operating characteristic curve or ROC curve. AUC provides an aggregate measure of performance across all possible classification thresholds. Subsequently, the predicted monthly *Ae. albopictus* occurrence maps were generated.

The following are briefs of the k-medoids clustering. k-medoids is a classical partitioning clustering technique that clusters a dataset of objects into k groups known a priori [35]. The k-medoid clustering applied most extensively is the Partitioning Around Medoids (PAM) algorithm [35], which was selected for the present study. The method initially selects k data points as the medoids, and the points represent different clusters. Each data point is associated with the closest medoid through a stepwise method to minimize the cost of the configuration, i.e., sum of distances of points to their medoid. Briefly, for each medoid and non-medoid data point, swap medoid with non-medoid, and re-compute the cost. If the total cost of the configuration decreases, then continue, otherwise undo the swap. The process continues until all data points are assigned to clusters. In the study, we knew that *Ae. albopictus* occurred all-year-around in tropical China, in regions such as Hainan Island; however, one may observed adult *Ae. albopictus* only in summer from June to September in northern China, in regions such as in Liaoning province (Additional file 2: Table S1, Additional file 3: Table S2 and Additional file 4: Figure S1). The initial k value was set as 12, and values including 11, 10, 9, and 8 were also examined. We used data points with known *Ae. albopictus* occurrence months as training data then used clustering results to assign occurrence months to each record in cases where they were missing due to a lack of longitudinal observations.

The machine learning classification and regression tree (CART) analysis was used to predict the monthly/seasonal distribution of *Ae. albopictus*. The binary values, 0 and 1, representing ‘presence’ and ‘absence’, respectively were assigned to each month for all sampling points based on field observation or the results of k-medoids clustering. The binary variables were then used for CART modeling as the dependent variables, while environmental variables were the independent variables. We have adapted the commonly used 10-fold cross-validation rule in the present study [37, 38]. Consequently, 10 prediction models were produced for each month. The training sets were used to grow the tree and the testing sets were used for cross-validation to prune the tree. We set a minimum data points of five for all terminate nodes. Since *Ae. albopictus* was only present in a small area in southern China in winter (December–February) and absent in a small area in northern China in summer (July–September), the stratification (i.e., rearrangement of the data) 10-fold cross-validation method was used to ensure that each fold was an appropriate representative of the whole to minimize sampling bias induced by 10-fold random selection [37, 38].

Once the models were established, climatic-environmental suitability maps were generated for each month based on climatic surface, climatic zone, and regional environmental data. Suitability was predicted as the average predicted suitability probability of the 10 models developed during the 10-fold cross-validation modeling process, and spatial resolution was 30 arcsec or approximately 1 km^2^ spatial resolution.

All data analyses were conducted and maps generated using the open-source programming language R v3.3.2 (R Foundation for Statistical Computing, Vienna, Austria). For *k*-medoids clustering, we used the *pam* method of the *cluster* package; for raster image reading and risk mapping, we used the *raster* and *crop* methods within the *rasterImage* and *sp* packages; and for regression tree modeling, we used the *ctree* and *rpart* methods within the *rpart*, *party*, and *caret* packages.

## Results

### Updated *Ae. albopictus* distribution map in China

**Fig. 1:**
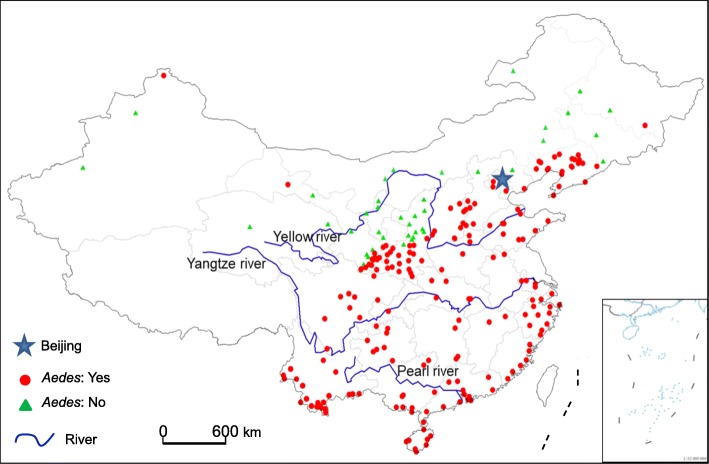
Map of *Aedes albopictus* surveillance sites in China. Bottom right box: South China Sea islands

Two hundred and forty-two sampling locations were included in the present study (Fig. 1). *Ae. albopictus* was recorded in 200 sites and no *Ae. albopictus* were observed in the rest of the 42 sites. Only four provinces had ongoing systematic surveillance programs, including two southern provinces, Zhejiang and Hainan Island, representing the subtropical-tropical areas; one southwestern province, Guizhou, which is a high-elevation mountainous area; and Shaanxi province, representing northern China, and potentially the northern distribution margin of *Ae. albopictus* in China (Fig. 1, Additional file 4: Figure S1) [39–42]. Data records included 31 sampling sites (county or city) in Shaanxi province, in which *Ae. albopictus* occurred in 20 sites (Fig. 1, Additional file 4: Figure S1). Gansu province, in the semiarid and arid area of northwestern China, had 22 sampling sites, of which 14 had *Ae. albopictus* (Fig. 1). No *Ae. albopictus* were observed in the Ningxia Hui Autonomous Region, the Inner Mongolia Autonomous Region, or Qinghai province, which are all located in the semiarid, arid or Qinghai-Tibetan Plateau temperate areas (Fig. 1). No sampling has been conducted previously in the Tibet Autonomous Region, and very few collections have been carried out in Qinghai province, and the Xinjiang Uygur Autonomous Region; the three provinces represent some of the provinces with the largest land areas and the harshest climatic and environmental conditions in western China (Additional file 4: Figure S1). Most of the *Ae. albopictus* positive sites in the present study are in areas where ,the species have been found before, and the areas are considered to have suitable climatic conditions (Fig. 1, Additional file 4: Figure S1) [20, 22, 23, 32]. Notably, however, some more recent reports of *Ae. albopictus* are far outside the conventional suitable climate ranges including a site in Heilongjiang province in northeastern China, which borders the Democratic People’s Republic of Korea, a site in the far west of Gansu province, in the desert city of Jiayuguan, and a site in northern Xinjiang, which borders Russia, Mongolia and Kazakhstan (Fig. 1). There are also several *Ae. albopictus* positive sampling sites in northern Liaoning province, in which the vector has never been reported previously, in addition to two sites on the east slope of the Qinghai-Tibet Plateau in western Sichuan province (Fig. 1, Additional file 4: Figure S1).

### Seasonal risk modeling

**Fig. 2:**
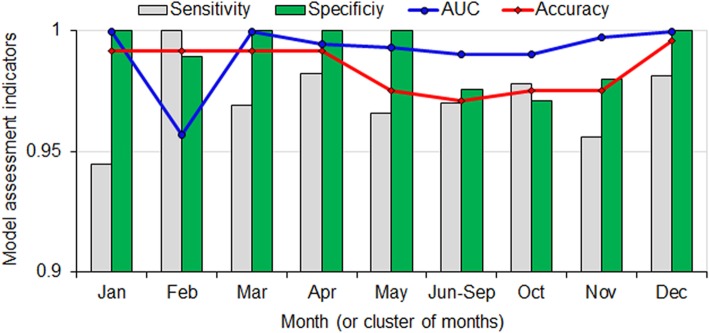
Model sensitivity, specificity, accuracy, and area under curve based on months

Climatic and environmental similarity analysis results suggested that the observational data points could be clustered into nine clusters because all the *Ae. albopictus* occurrence sites corresponded to the June–September period; therefore, regression trees were developed for 9 months or clusters of months, with June–September considered one cluster. Figure 2 illustrates the sensitivity, specificity, and accuracy of the models, in addition to the AUC (Fig. 2, Additional file 5). Using a cutoff yield the optimal accuracy for each month or cluster of months. The average model accuracy was 98.4% (range: 97.1–99.9%) and the average AUC was 99.1% (range: 95.7–99.9%). The sensitivity rate was 97.2% on average (range: 94.4–98.2%), with the lowest value in January and the highest in April. The specificity rate was 99.1% on average (range: 97.1–100%).

### Seasonal risk mapping

**Fig. 3:**
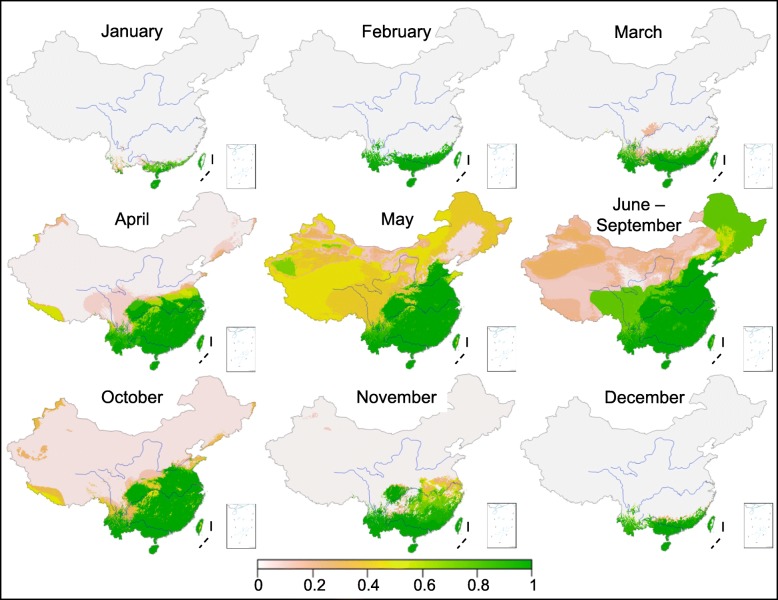
Maps of *Aedes albopictus* risk probabilities based on months or clusters of months. Blue curves are the major rivers in China from north to south: Yellow (Huanghe), Yangtze (Changjiang), and Pearl (Zhujiang) rivers. There was no prediction for the South China Sea islands; therefore, South China Sea islands are not showing displayed in the graph (see Fig. 1, bottom right box)

The model unequivocally predicts that in the winter months (December–February) *Ae. albopictus* will be observed only in southern China, as depicted in the risk maps (Fig. 3). The major expansion of the range of *Ae. albopictus* adults begins in April, when the predicted occurrence covers most of the areas south of the Yellow River (Fig. 3). By May, it covered all of China, including a few places in northwestern China, although risk probabilities are low in the northeast and southwest regions (Fig. 3). In June–September, the major dengue fever transmission season in China, the model predicts the greatest range of climate and environmental suitability. The most notable areas are included northeastern China, and the eastern slope of the Qinghai-Tibet Plateau in southwestern China (Fig. 3), which have been predicted in previous studies to have unsuitable climates. Predicted ranges decreased considerably by October compared to June–September, when the risk map is similar to the map of April. In November, *Ae. albopictus* adults remained active across most regions south of the Yangtze River and in a small portion of the areas north of the Yangtze River (Fig. 3).

## Discussion

*Ae. albopictus* has extended its range globally due to its capacity to adapt to the environment and climate change [1–5]. The exploration of the environmental factors influencing its distribution range in China could facilitate the formulation of effective monitoring and risk assessment programs, since new dengue fever outbreaks are a threat to public health. Indeed, recent dengue fever epidemics have occurred in different regions ranging from Guangzhou, in southern China, in 2014, to Yuzhou in Henan province, which is on the south bank of the Yellow River in central China, 1400 km straight north of Guangzhou, in 2013 [43]. Almost all the recent epidemics were caused solely by *Ae. albopictus* [6–9, 43]. The 2013 dengue fever epidemic in Yuzhou, initiated by an imported index case, pointed to the suitability of the local environment-climate in central China. The danger lies in the fact that imported cases have been reported all over China [44]; therefore, there is a need to update the vector distribution maps and reassess environmental suitability based on the updated distribution data. Although our analysis introduced new sampling sites in China, we anticipated *Ae. albopictus* distributional potential similar to that reported in the previous studies [20, 22]. The five most notable sites included two in northwestern China (Jiayuguan City in Gansu and Beitun City in Xinjiang) and one in northeastern China (Ning’an County in Heilongjiang), and two sites in Jiulong and Lixian counties in western Sichuan province, which are located at high altitude (close to 3000 m above sea level) on the east slope of the Qinghai-Tibetan Plateau (Fig. 1). Using environmental data from the new sites, the present study generated a new risk map with risk ranges extended considerably in summer, particularly in northeastern China in areas bordering Russia and on the eastern slope of the Qinghai-Tibetan Plateau in southwestern China. The predicted regions with suitable climates from May to September extended beyond the areas predicted substantially even in other recent studies [22, 32]. Whether or not the expansion in the *Ae. albopictus* population range is linked to global climate change or the biological adaptation of vectors requires further study.

Based on the updated distribution data, the present study predicted that *Ae. albopictus* could occur seasonally in semiarid or even arid areas, as long as temperature and precipitation conditions are favorable, which suggests a need to consider seasonal climate suitability as opposed to focusing on specific annual periods. In addition to temperature, water availability influences mosquito distribution [45]. In most temperate areas of China, precipitation is highly seasonal and temperatures are usually high in summer [21, 22]. The short summers, relatively high temperature, and seasonal rainfall in northern China could facilitate the establishment of *Ae. albopictus* populations in the region, which has occurred in Japan in regions at similar latitudes and in the northeastern United States [46, 47]. With regard to the occurrence of *Ae. albopictus* in semiarid and arid areas, the key examples are Pakistan and Saudi Arabia, where dengue fever outbreaks have been reported previously [46, 47].

Other *Ae. albopictus* risk areas of interest are the southwestern international border areas and the eastern slope of the Qinghai-Tibetan Plateau. Could the environmental conditions in such regions facilitate *Ae. albopictus* population establishment? In the present study, five *Ae. albopictus* occurrence sites had elevations > 2000 m above sea level, with the highest at approximately 2900 m. Dengue fever epidemics and *Ae. albopictus* have been reported in northern Pakistan, not far from the China-Pakistan border [46, 47]. In addition, dengue fever epidemics and both *Ae. albopictus* and *Ae. aegypti* have been reported in Nepal [48–50], which lies on the south slope of the Himalaya mountain range, also bordering southwestern China. Such studies indicate that vectors of tropical infectious diseases could adapt to the environmental conditions in the low altitude section of the Qinghai-Tibetan Plateau and could even transmit the diseases there. Again, the influence of global climate change on such dynamics is a subject for further study.

The present study has some major limitations. The model applied climatic data from 1971 to 2000, which may have limited its capacity to predict the recent expansion of *Ae. albopictus* ranges, although WorldClim data has been widely used for the prediction of the impact of global change on the expansion of *Ae. albopictus* ranges [13, 19, 31, 34]. Since climate change has intensified over the past two decades (http://www.ncdc.noaa.gov/cag/), incorporating the most recent data could yield an even more substantial *Ae. albopictus* range expansion or late-year (late fall or early winter) activity predictions [18, 31]. For example, the present study predicted that *Ae. albopictus* adult activity would be restricted to the south of the Yellow River by October and to the south of the Yangtze River by November.

A previous study conducted in Shandong province, along the banks of the Yellow River, trapped *Ae. albopictus* outdoors in November [51], which our model predicted as ‘absence’ with zero probability. This could be due to 1) old climatic data used in the modeling activity; 2) annual climate variability; or 3) real temperature increase or climate change, which warrant further investigation. Another limitation of the present study is the lack of human distribution data [52–54]. While environmental factors influence vector population growth, human population distribution influences the transmission of disease [54, 55]. In the arid areas of northern and northwestern China and in numerous parts of the Qinghai-Tibetan Plateau, the human population distribution is sparse; therefore, potential dengue fever risks are low, particularly in the frigid area on top of the plateau. However, how climate and environmental factors interact with human distribution in landscapes and biological competitors, and their combined influence on *Ae. albopictus* population establishment and distribution require further investigation. Since the sampling sites in the present study were point data, further analyses, such as spatial extrapolation studies, are required, so that expansion ranges and increased risks in populations can be estimated in combination with population density maps. A further prospect would be analyzing local climate trends in the past and linking them with observed dengue fever outbreaks, which would be more appealing from a public health perspective than simply focusing on vector distribution ranges [1–5]. Furthermore, when mapping the distribution and seasonality of *Ae. albopictus* and dengue fever outbreaks in China, it could be more appropriate to use only meteorological data from stations in China and international stations near the borders. This warrants further explorations and it is the objective of our ongoing project.

Finally, systematic sampling, i.e., good spatial coverage and sufficient longitudinal follow-ups, is key for the determination of the distribution ranges conduciveness of climatic conditions for *Ae. albopictus*. Currently, systematic survey data on *Ae. albopictus* are only available for four provinces in China [39–42]. Although China’s Center for Disease Control and Prevention has implemented a cross-country surveillance system and conducted longitudinal surveys, the spatial coverage is too coarse for a comprehensive nationwide evaluation of *Ae. albopictus* distribution [6, 23]. June–September is an important season for mosquito population dynamics monitoring, based on the results of this study. We emphasize that both spatial coverage and sampling seasons are essential for determining the northern geographic margins of distribution of the vector.

## Conclusions

Both climatic and environmental factors both influence *Ae. albopictus* distribution in China. The current predicted seasonal distribution of *Ae. albopictus* in China may extend well beyond the ranges predicted previously, particularly the northern limit, due to either global climatic change or the adaptation of biological vectors. Considering the sustained movement of dengue fever epidemics northward in central and eastern China, the timely monitoring of *Ae. albopictus* populations and their expansion, the assessment, and frequent reassessment of epidemic risks in such areas, and the assessment of risks based on recent dengue fever outbreaks in China, particularly in central and northern China, are crucial for epidemic preparedness and disease control, particularly in the wake of global climate change.

## Supplementary information

**Additional file 1.** Multilingual abstracts in the five official working languages of the United Nations.

**Additional file 2: Table S1.** Reference list for *Aedes albopictus* surveillance records.

**Additional file 3: Table S2.** List of *Aedes albopictus* surveillance locations used in the present study.

**Additional file 4: Figure S1.** Map of China with provinces and their boundaries. Bottom right box: South China Sea islands.

**Additional file 5: Figure S2.** Graphs of receiver operating characteristic curves (ROCs) for each month or cluster of months.

## Data Availability

The data sets supporting the results are included within the article and Additional file 1.
