# Supplementary material for: Seasonality modeling of the distribution of Aedes albopictus in China based on climatic and environmental suitability
Source: Infect Dis Poverty. 2019 Dec 3;8:98. doi: 10.1186/s40249-019-0612-y (PMC6889612; doi:10.1186/s40249-019-0612-y)
Supplement: Supplementary file 1 — Additional file 1. Multilingual abstracts in the five official working languages of the United Nations. [file 40249_2019_612_MOESM1_ESM.pdf]

Translation of the abstract into the five official working languages of the United Nations

النمذجة الموسمية لتوزيع *بعوض النمر الآسيوي* في الصين على أساس ملاءمة البيئة المناخية

زويلي تشنغ، ديبين تشونغ، يولان هو، غوفا زهو

ملخص

معلومات عامة: *يُعدّ بعوض النمر* الآسيوي نوعاً من البعوض شديد الغزو وناقلاً رئيسياً لعديد من الأمراض الفيروسية . يعود سبب العديد من حالات تفشي حمى الضنك التي حدثت مؤخرًا في الصين لهذا البعض وحده. *يعد وضع الخرائط* لنطاقات التوزيع المحتملة لبعوض النمر الآسيوي أمراً مهماً للاستعداد للوباء ورصد أعداد البعوض المسببة للأمراض لمكافحة المرض. يُعتبر المناخ عاملاً رئيسياً يؤثر على توزيع البعوض. على الرغم من الدراسات الميدانية التي تشير إلى التباينات السكانية الموسمية لبعوض النمر الآسيوي، إلا أنه لم يتم إجراء سوى القليل من عمل النماذج لتحليل كيفية تأثير الظروف البيئية على موسمية بعوض *النمر الآسيوي*. تهدف هذه الدراسة إلى بناء نموذج تنبؤي يعتمد على الملاحظات المتاحة والبيانات المناخية والبيئية وطرق التعلم الآلي للتنبؤ بالتوزيع الموسمي المحتمل لبعوض النمر الآسيوي في الصين.

الطرق: قمنا بجمع بيانات مراقبة شاملة محدّثة في الصين، وبالتحديد أكثر جمعنا السجلات من هامش التوزيع الشمالي لبعوض النمر الآسيويتم تعيين متوسطات بيانات مناخية طويلة الأجل لجميع السجلات (1970-2000) بالاستناد إلى النسخه الثانيه من نظام ورلد كلايمت. تم تطوير نماذج شجرة الانحدار للتعلم الآلي باستخدام طريقة تحقق متقابل بعشرة أضعاف للتنبؤ بنطاقات التوزيع الموسمية (أو الشهرية) المحتملة *لبعوض النمر الآسيوي* في الصين بدقة عالية استنادًا إلى الظروف البيئية. تم تقييم النماذج باستخدام الحساسية والنوعية والدقة والمساحة تحت المنحنى. تم استخدام بيانات النسخة الثانية من ورلد كلايمت ومناطق المناخ والبيئة لإنتاج أسطح تنبؤ بيئية مواتية (احتمالية). تم إنشاء الاحتمالات المتوقعة بناءً على متوسط النماذج العشرة.

النتائج: بين ١٩٩٨ و ٢٠١٧

تم ملاحظة بعوض النمر الآسيوي في ٢٠٠ منطقة من أصل ٢٤٢ تم إجراء مسح فيها. بالإضافة إلى ذلك، يقع ما لا يقل عن ١٥ موقع جديد يمكن العثور فيه على *بعوض النمر الاسيوي* خارج النطاقات المحتملة التي تم التنبؤ بها باستخدام النماذج في وقت سابق. كان متوسط الدقة ٩٨,٤٪ (النطاق: ٩٧,١٪-٩٩,٥٪) ، ومتوسط المساحه تحت المنحنى كانت ٩٩,١٪ (النطاق: ٩٥,٦٪ - ٩٩,٩٪) اقتصر انتشار *بعوض النمر الاسيوي* المتوقع خلال الشتاء (ديسمبر - فبراير) اقتصر على منطقة صغيرة شبه استوائية من الصين، وتم توقع أن *بعوض النمر الآسيوي* ينتشر في شمال الصين فقط خلال موسم الصيف القصيرة (عادة من يونيو إلى سبتمبر). يمكن أن تصل مناطق التوزيع المتوقعة في الصيف إلى شمال شرق الصين على الحدود مع روسيا والجزء الشرقي من هضبة تشينغهاي تيبيت في جنوب غرب الصين. يمكن أن يبقى *بعوض النمر الآسيوي* نشطاً في المناطق الممتدة من وسط إلى جنوب الصين في أكتوبر ونوفمبر.

الاستنتاجات: المناخ والظروف البيئية هي العوامل الرئيسية التي تؤثر على التوزيع الموسمي لبعوض النمر الاسيوي في الصين. يمكن أن تصل المناطق التي من المتوقع أن تستضيف *بعوض النمر الآسيوي* موسمياً في هذه الدراسة إلى شمال شرق الصين والمنحدر الشرقي من هضبة تشينغهاي تيبيت. تقدم نتائجنا أدلة جديدة وتقترح توسيع أنشطة مراقبة أعداد وأنشطه ناقلات الأمراض وإعادة تقييم احتمالات خطر الوباء بانتظام

Translated from English version into Arabic by Hamid Alahmari, revised by Muhannad Albayk Jaam, through

TRANSLATORS WITHOUT BORDERS

基于气候-环境适应性模型预测白纹伊蚊在中国季节分布

郑学礼，钟代斌，何玉兰，周国法

摘要

引言：白纹伊蚊是一种高度入侵性的蚊种，为多种病毒病原体的主要传播媒介。最近中国发生的许多登革热疫情都是由该种蚊虫传播引起的。模型预测白纹伊蚊的潜在分布范围，在疾病流行预警、媒介种群监测、控制疾病方面起着关键作用。气候是影响白纹伊蚊分布的一个关键因素。虽然有现场研究报告白纹伊蚊季节种群变化，但鲜见采用模型分析环境条件如何影响白纹伊蚊的季节性变化的研究。本研究的目的是基于现有的观测、气候和环境

资料，建立模型，用机器学习方法预测白纹伊蚊在中国的潜在季节性分布范围。
方法：本研究收集回顾白纹伊蚊在中国的监测记录，尤其是中国北方地区分布边界的记录。基于 WorldClim 2.0 数据集，将 1970–2000 年间所有的气候数据资料进行平均分配。采用一个 10 倍交叉验证方法根据环境条件建立发展机器学习回归树模型，以预测白纹伊蚊在中国的潜在季节性（或月份）分布范围。采用曲线下面积（AUC）评估模型的敏感性、特异性和精确性。用 WorldClim 2.0 和气候、环境资料，产生环境概率预测面积。基于 10 个模型平均值生成预测概率。
结果：1998–2017 年间，调查 242 个位点，其中 200 个调查点存在白纹伊蚊。另外，发现 15 个新的白纹伊蚊发生位点，它们位于以前采用模型预测的潜在范围之外。预测的平均精确性是 98.4%（97.1～99.5%），平均 AUC 是 99.1%（95.6～99.9%）。预测白纹伊蚊在冬季（12 月～2 月）局限于中国部分亚热带-热带区域，预测中国北方地区白纹伊蚊仅见于较短的夏季（常为 6 月～9 月）。预测白纹伊蚊的夏季分布区可以到达中国东北部与俄罗斯接壤的地区和中国西南部青藏高原东部地区。在 10 月和 11 月，白纹伊蚊仍活跃在中国中部和南部的广阔地区。
结论：气候和环境条件是影响白纹伊蚊在中国季节性分布的关键因素。本研究预测白纹伊蚊潜在季节分布范围可达到中国东北部和青藏高原东坡。本研究结果提示，需扩大系统性媒介种群监测，定期重新评估疾病流行的潜在风险。

Translated from English version into Chinese by Xueli Zheng

Modélisation de la saisonnalité de la distribution du *Aedes albopictus* en Chine basé sur la compatibilité du climat et de l'environnement

Xueli Zheng, Daibin Zhong, Yulan He, Guofa Zhou

Résumé
Contexte: le moustique *Aedes albopictus* fait partie des espèces de moustiques très invasives et est un vecteur majeur de nombreux agents pathogènes viraux. En Chine, de nombreuses épidémies récentes de dengue ont été causées uniquement par ce vecteur. Afin d'être mieux préparé en cas d'épidémies et afin de surveiller les populations de vecteurs pour contrôler la maladie, il est essentiel de cartographier la distribution potentielle du *Aedes albopictus*. Le climat est un facteur déterminant de la répartition de l'espèce. Malgré des études de terrain indiquant des variations saisonnières de la population, très peu de travaux de modélisation ont été réalisés dans le but d'analyser l'influence des conditions environnementales sur la saisonnalité du *Ae. albopictus*. Le but de cette étude est de construire un modèle prédictif basé sur les observations, les données climatiques et environnementales disponibles ainsi que des méthodes d'apprentissage automatique pour prédire la distribution saisonnière potentielle du *Ae. albopictus* in China.
Méthodes: nous avons collecté en Chine des données de surveillance complètes et actualisés, en particulier des enregistrements de la marge de distribution nord du *Ae. albopictus.* Tous les enregistrements ont été affectés à des moyennes de données climatiques à long terme (1970-2000) basées sur le WorldClim 2.0. Des modèles d'arbres de régression d'apprentissage automatique à haute résolution ont été développés à l'aide d'une méthode de validation croisée par 10 afin de prédire les aires de distribution saisonnières (ou mensuelles) potentielles du *Ae. albopictus* en Chine en fonction des conditions environnementales. Les modèles ont été évalués à l'aide de la sensibilité, de la spécificité, de la précision et de l'aire sous courbe (ASC). Les données du WorldClim 2.0 et des zones climat-environnement ont été utilisées pour créer des surfaces de prédiction de la conductivité environnementale (probabilité). Les probabilités prédites ont été générées en fonction de la moyenne des 10 modèles.
Résultats: entre 1998 et 2017, *Ae. albopictus* a été observé dans 200 des 242 localités étudiées. De plus, au moins 15 des nouveaux sites d'occurrence du *Ae. albopictus* se situaient en dehors des aires potentielles prédites à l'aide de modèles. La précision moyenne était de 98,4% (intervalle : 97,1 à 99,5%) et l'ASC moyenne de 99,1% (intervalle : 95,6 à 99,9%). La

répartition prévisionnelle du *Ae. albopictus* en hiver (décembre à février) était limitée à une petite région subtropicale/tropicale de la Chine. *Ae. albopictus* ne devait être présent dans le nord de la Chine que pendant la courte saison estivale (généralement de juin à septembre). Les zones de répartition prévisionnelles en été peuvent atteindre le nord-est de la Chine limitrophe de la Russie et la partie est du plateau Qinghai-Tibet, dans le sud-ouest de la Chine. *Ae. albopictus* pourrait rester actif en octobre et en novembre dans les régions en expansion du centre et du sud de la Chine.
Conclusions: Les conditions climatiques et environnementales sont des facteurs clés influant sur la distribution saisonnière du *Ae. albopictus* en Chine. Cette étude montre que les zones susceptibles d'héberger de façon saisonnière *Ae. Albopictus* peuvent atteindre le nord-est de la Chine et le versant est du plateau Qinghai-Tibet. Nos résultats présentent de nouvelles preuves et suggèrent l'extension des activités de surveillance systématique de la population de vecteurs et la réévaluation régulière du potentiel de risque épidémique.

Translated from English version into French by Laetitia Stievenart, revised by Emilie Rigault Fourcadier, through

TRANSLATORS
WITHOUT BORDERS

Моделирование сезонности распространения *Aedes albopictus* в Китае на основе климатической и экологической приемлемости

Сюэли Чжэн (Xueli Zheng), Дайбинь Чжун (Daibin Zhong), Юйлань Хэ (Yulan He), Гофа Чжоу (Guofa Zhou)

Реферат

Общие сведения: *Aedes albopictus* — это высокоинвазивный вид комаров и основной переносчик многочисленных вирусных патогенов. Возникновение многих недавних вспышек лихорадки денге в Китае обусловлено исключительно этим переносчиком. Картирование потенциальных ареалов распространения *Ae. albopictus* имеет решающее значение для обеспечения готовности к эпидемиям и мониторинга популяций переносчиков инфекции в целях борьбы с ними. Климат является ключевым фактором, влияющим на распространение вида. Несмотря на результаты полевых исследований, указывающих на сезонные колебания численности популяции, было проведено недостаточно работ по моделированию для анализа влияния климатических условий на сезонность *Ae. albopictus*. Целью данного исследования является построение модели прогнозирования на основе имеющихся наблюдений, климатических и экологических данных и методов машинного обучения для прогнозирования возможного сезонного распределения *Ae. albopictus* в Китае.
Методы: Мы собрали обширные данные наблюдений в Китае, в частности записи с северной границы распространения *Ae. albopictus*. Всем записям были присвоены средние долгосрочные (1970-2000 гг.) климатические данные, основанные на WorldClim 2.0. Модели регрессионного дерева машинного обучения были разработаны с использованием 10-кратного метода перекрестной валидации для прогнозирования возможного сезонного (или месячного) распределения *Ae. albopictus* в Китае с высоким разрешением в зависимости от условий окружающей среды. Модели оценивались с использованием чувствительности, специфичности, точности и площади под кривой (ППК). Данные WorldClim 2.0 и климатически-экологических зон были использованы для получения поверхностей прогнозирования проводимости (вероятности) окружающей среды. Прогнозируемые вероятности были получены на основе средних значений по 10 моделям.
Результаты: В период с 1998 по 2017 гг. *Ae. albopictus* наблюдали в 200 из 242 обследованных населенных пунктов. Кроме того, по меньшей мере 15 новых областей обитания *Ae. albopictus* лежат за пределами потенциальных

диапазонов, которые были предсказаны с использованием ранее построенных моделей. Средняя точность составила 98,4% (диапазон: 97,1-99,5%), а средняя ППК - 99,1% (диапазон: 95,6-99,9%). Прогнозируемое распространение *Ae. albopictus* зимой (декабрь-февраль) ограничивалось небольшой субтропической и тропической зоной Китая, при этом появление *Ae. albopictus* в северном Китае прогнозировалось только в короткий летний сезон (обычно в июне-сентябре). Прогнозируемые районы распространения в летний период могут охватывать северо-восточную часть Китая, граничащую с Россией и восточной частью Цинхай-Тибетского нагорья на юго-западе Китая. *Ae. albopictus* могут проявлять активность в обширных районах от центра до юга Китая в октябре и ноябре.

Выводы: Климатические и экологические условия являются ключевыми факторами, влияющими на сезонное распространение *Ae. albopictus* в Китае. Районы, в которых по прогнозам данного исследования потенциально могут обитать *Ae. albopictus* сезонно, могут охватить северо-восточный Китая и восточноый склон Цинхай-Тибетского нагорья. Полученные нами результаты позволяют получить новые данные и предполагают расширение деятельности по систематическому мониторингу популяции переносчиков и регулярную повторную оценку уровня эпидемического риска.

Translated from English version into Russian by Michael Orlov, revised by Veronika Demeshchyk, through

TRANSLATORS
WITHOUT BORDERS

Construcción basada en la adecuación clima-medio ambiente de modelos de estacionalidad para la distribución del *Aedes albopictus* en China

Xueli Zheng, Daibin Zhong, Yulan He, Guofa Zhou

Resumen

Antecedentes: *El Aedes albopictus*es una especie de mosquito altamente invasiva y un importante portador de numerosos patógenos virales. Muchos brotes recientes de dengue en China han sido causados únicamente por este portador. Hacer un trazado de los márgenes de distribución potencial del *Ae. albopictus* es crucial para poder hacer frente a las epidemias y vigilar a las poblaciones portadoras para el control de enfermedades. El clima es un factor clave que influye en la distribución de la especie. A pesar de los estudios de campo que indican variaciones estacionales de población, no se ha trabajado mucho en construir modelos para analizar cómo las condiciones del medio ambiente influyen en la estacionalidad del *Ae. albopictus*. El propósito de este estudio es construir un modelo predictivo basado en las observaciones disponibles, los datos climáticos y medioambientales y los métodos de aprendizaje automático, para predecir la potencial distribución estacional del *Ae. albopictus* en China.

Metodología: Recopilamos datos exhaustivos y actualizados de seguimiento en China, en particular registros sobre el margen de distribución del *Ae. albopictus en el norte.* A todos los registros se les asignaron las medias de datos climáticos a largo plazo (1970-2000) en función del WorldClim 2.0. Se desarrollaron modelos de árbol de regresión por medio de un método de validación cruzada de 10 iteraciones para predecir los rangos potenciales de distribución estacional (o mensual) del *Ae. albopictus* en China, en alta resolución y según las condiciones del medio ambiente. Se evaluaron los modelos teniendo en cuenta la percepción, especificidad, precisión y el área bajo la curva (AUC). Se utilizaron el WorldClim 2.0 y datos de zonas climáticas y medioambientales para generar superficies de predicción de favorabilidad (probabilidad) medioambiental. Se generaron probabilidades predictivas según la media de los diez modelos.

Resultados: Entre 1998 y 2017, el *Ae. albopictus* fue observado en 200 de las 242 localidades investigadas. Al menos 15 de las nuevas ubicaciones donde está presente el *Ae. albopictus* se encuentran fuera de los márgenes potenciales pronosticados anteriormente utilizando los modelos. La precisión media fue de 98,4 % (rango: 97,1-99,5 %), y la media fue de 99,1 % (rango: 95,6-99,9 %). La distribución predictiva del *Ae. albopictus* en invierno (diciembre-febrero) se limitó a una pequeña zona de China, y se pronosticó que el *Ae. albopictus* se encontraría en el norte de China solo durante la corta estación veraniega (habitualmente junio-septiembre). Las áreas de distribución predictiva en verano pueden alcanzar el nordeste de China en la frontera con Rusia y la parte oriental de la meseta de Qinghai-Tíbet en el sudoeste de China. El *Ae. albopictus* podría permanecer activo en áreas expansivas entre el centro y el sur de China en octubre y noviembre.

Conclusiones: Las condiciones climáticas y medioambientales son factores clave que influyen en la distribución estacional del *Ae. albopictus* en China. Las áreas en las que se prevé que pueda encontrarse estacionalmente el *Ae. albopictus* en el presente estudio alcanzan la China del nordeste y la ladera este de la meseta de Qinghai-Tíbet. Nuestros resultados presentan nuevas pruebas y sugieren la expansión de las actividades sistemáticas de control de la población de portadores y la realización frecuente de nuevas evaluaciones del potencial de un riesgo epidémico.

Translated from English version into Spanish by Pedro Jesús Quintana, revised by Maria Paula Gorgone, through

TRANSLATORS
WITHOUT BORDERS
