# Supplementary material for: Seasonality modeling of the distribution of Aedes albopictus in China based on climatic and environmental suitability
Source: Infect Dis Poverty. 2019 Dec 3;8:98. doi: 10.1186/s40249-019-0612-y (PMC6889612; doi:10.1186/s40249-019-0612-y)
Supplement: Supplementary file 2 — Additional file 2: Table S1. Reference list for Aedes albopictus surveillance records. [file 40249_2019_612_MOESM2_ESM.docx]

Supplement

Table S1. References list of field investigations

1. Fan J, Cao H, Zhou Y-B, Jiang Q-W. Study of mosquito species, density and seasonal changes in Huangpu District of Shanghai from 2008 to 2015. Shanghai J Prev Med 2016, 28(8): 521-4.
2. Wang SZ, Xu RQ, Xu YX, Ren WJ, Jiang LY, Hu JG, Cai EM. Surveillance on dengue vector and breeding habit of vector in Shanghai area. Chinese J Vector Biol Control 2003, 14(2): 124-6.
3. Zhang H, Zi D, Gong Z. The epidemiological survey of dengue fever in Yunnan Province, China. Endemic Dis Bull 1999, 14(3): 50-4.
4. Wang P-Y, Wu C, Zhang C-L. Survey of the transmission vectors of dengue fever in some areas in Yunnan Province. China Trop Med 2006, 6(11): 1933-4.
5. Wang G, Zheng Z, Dong Y-D, Zhao T-Y. Mosquito communities in boarder areas between China and Burma. Acta Parasitol Med Entomol Sin 2011, 18(4): 216-9.
6. Guo X-F, Wang P-Y, Zhou C-L, Dong L-M, Zhou H-N. Relationship between the seasonal nuctuation of mosquito populations and the epidemic of *Japanese encephalitis* in Jinghong County, Yunnan Provence. Acta Parasitol Med Entomol Sin 2011, 18(4): 198-203.
7. Bao J-Z. Surveys of mosquitoes in Chuxiong prefecture, Yunnan province. China’s Rural Health 2016, 4: 39-40.
8. Fan Z⁃H, Wei R⁃Q, Li L⁃J, Jin Z⁃H, Li J, Guo X⁃F, Wang Y⁃B1, Zhang D, Jiang X⁃Y, Yang M⁃D. Investigation of Aedes mosquito distribution in the central Yuxi city. Chin J Vector Biol & Control 2016, 27(6): 614-6.
9. Chen H, Chen H-B. Comparative studies on hydrolysable amino acids in geographic strains of *Aedes albopictus* from Hainan and Beijing. Guizhou Med 2002, 26(3): 227-9.
10. Kong F, Wang H, Song H, Zheng J, Kong Y, Kong F. Survey of Aedes and its control at Confucious Forest in Qufu. Chin J Vector Bio & Control 2001, 12(3): 195-6.
11. Jiang H-R, Lin S-X, Wang Y, Zhao Y-J. The investigation and control strategy on important vectors in Qingdao. Chin J Vector Bio & Control 2007, 18(5): 369-71.
12. Du J, Liu C, Wu X, Jin C. Study on the biology of *Aedes albopictus* in southern Shangdong. Med Vet Prev 2001, 17(11): 561-2.
13. Zhang L, Li W, Li K, Fan Z, Huang W, Chen Y, Cai J, Yue F. Study on the vector and dengue virus infection in Guangzhou in 2006. Chin J Vector Bio Control 2007, 18(5): 414-6.
14. Lin H-S, Zhang W, Wang S-W, Chen H-S. Analysis and monitoring of dengue fever vector in Leizhou city in 2006. Chin J Vector Bio Control 2008, 19(5): 464-6.
15. Xia S-L, Wang T, Shu B, Zhang R-H, Wu H-D, Luo L. Analysis on results of monitoring dengue fever and investigation on dengue vector during 2005-2007 in Zhongshan City. J Trop Med 2008, 8(6): 619-21.
16. Gao SZ, Luo XM, Wu BY. Serologic surveillance of dengue fever in Zhongshan City during the period of 2004~2005. China Tropical Medicine 2006, 6(4): 600.
17. Jin L-Q, Guo Y. Investigation on the density and larval habitats of dengue vectors in Shantou, Guangdong Province. J Shantou University Med College 2007, 20(3):167-9.
18. Fan Z-F, Li W-J, Yang L-M, Li R-M. Study on the epidemiological characteristic and factor of dengue fever outbreaks in Yang Jiang City in 2001-2006. Chin Trop Med 2007, 7(3): 352-4.
19. Liang H, Li Z, Zeng X. Comparison of adult mosquito densities in different habitats in Hengqin. Chinese Frontier Health Quarantine 2002, 25(suppl): 59-61.
20. He J-P, Luo J-G, Yang M-L, Li Z-Q. Survey on seasonal growth of *Aedes albopictus* larva at Guangzhou Xinfeng Port. Inspection Quarantine Sci 2007, 17(4): 37-41.
21. Cai SW, Lin LF, Duan JH, Yin WX. *Aedes albopictus* insecide resistance status and management strategy in Guangdong Province. Chinese J Vector Biol Control 2006, 17(4): 274-6.
22. Wu L, Li M, Duan J. *Aedes* larval container index in Wenjin, Shenzhen from 2003.4-2004.3. Port Health Control 11(1): 28-30.
23. Ye H, Lan Q, Su S, Feng D. Monitoring *Aedes* mosquito populations in Dongxing port in 2011. Appl Prev Med 2013, 19(1): 55-7.
24. Tan M, Feng X-Y, Jiang J-H. Analysis on the Dengue vector surveillance results during 2002—2003 in Guangxi. Chinese J Hyg Insecticide & Equip 2004, 10(3): 154-6.
25. Lan C, Feng X, Liao G, Xiong Y, Jiang X, Nong L. Surveillance of disease vectors in Guangxi from 2005~2006. Applied Prev Med 2007, 13(6): 370-3.
26. Tang L, Zhang J, Ling T, He Y, Song J, Liu X, He Q. Surveillance of mosquitoes at Wuzhou port. Port Health Control 9(3): 23-26.
27. Tan Y, Feng XY. Dengue vector surveillance in Guangxi. ACTA Parasitologica et Medica Entomologica Sinica 2003, 10(2): 93-6.
28. Zheng Y-P, Li Y-L, Lou W-J, Zhang C-H, Gong X-Z. Investigation on mosquito kinds in 2004-2005 in Nanjing city. Chinese J Hyg Insecticide & Equip 2007, 13(2): 139-40.
29. Huang G, Li H-Y, Zhao Y, Wang X-M, Chang M, Wang Z-Q, Du M-T, Cao D-S, Chen C-H, Chen C-H, Guo Y-H, Liu Q-Y. The first investigation on distribution of Dengue vector *Aedes albopictus* in north and northwest of Hebei province. Chin J Vector Biol & Control 2007, 18(6): 450-2.
30. Wu F, Liu Q, Lu L, Wang J, Song X, Ren D. Distribution of *Aedes albopictus* (Diptera: Culicidae) in Northwestern China. Vector-Borne and Zoonotic Diseases 2011, 11(8): 1181-6.
31. Guo Y⁃H, Chen C⁃W, Zhu L, Liu X, Luo Y⁃D, Shen Y, Liu J⁃L, Liu Q⁃Y. Study on daily activity rhythm of *Aedes albopictus* in Yongcheng city. Chin J Vector Biol & Control 2016, 27(5): 484-6.
32. Wang W, Zhnag S, Meng F. The types and distribution of small mosquito larval habitats. Chin J Vector Bio & Control 2005, 16(3): 192.
33. Tan Q, Wang D, Han S. Surveillance of dengue vectors in Daishan county. Zhejiang Prev Med, 2014, 26(10): 1018-20.
34. Bai Y, Zhu G, Fan F, Xu R, Shao G, Chen Y. Investigation on the density and distribution of dengue vector *Aedes albopictus* in Ningbo. Chin J Vector Bio & Control 2007, 18(4):324-6.
35. Fu G-M, Yang T-C, Ren Z-Y. Population surveillance of mosquitoes in Zhejiang province in 2006. Chin J Hyg Insect ＆ Equip 2008, 14(2): 107-8.
36. Wu W-X, Jin Y-M, Sun L-Y, Zeng X-J, Su X-Y, Jia P-B, Li Z, Lao S-J. Analysis of results of sentinel monitoring of transmission vector of dengue fever in Hainan Province in 2006. China Trop Med 2007, 7(10): 1863-5.
37. Wang ZG, Wang SQ, Ono M, Masahi T, Yoshio T, Zeng LH, Wu QH, Lan XH, Guo RN, Liang QC, Li SG, Zhou XJ, Cai YR. Investigaton on *Ae. aegypti* and *Ae. albopictus* in the north-western part of Hainan Province. China Trop Med 2005, 5(2): 230-3.
38. Jin Y-M, Wu W-X, Sun L-Y, Su X-Y, Jia P-B, Li Z, Lao S-J, Nie S-F. Survey on the distribution of *Aedes* mosquitoes transmitting dengue fever of Hainan Province in 2007. China Trop Med 2008, 8(12): 2096-8.
39. Zhong H. Baseline investigation of disease vectors in Yueyang port. Frontier Health Quarantine 2001, 24(1): 26-8.
40. Huang X, He L, Li J. Investigation on species and seasonal change of mosquitoes in Chenzhou city. J Xiangnan Univ Med Sci 2012, 14(3): 29-31.
41. Huang M, Zhang P. Population surveillance and control of *Aedes albopictus* at Shuiguohu district, Wuhan. J Hubei College of TCM 2003, 5(3): 41.
42. Huang C, Chen M, Wang Q, Tang L. Research on the dengue vectors in Fuzhou in 2004. Guangxi Prev Med 2005, 11(4): 217-218.
43. Chen Y, Wang J, Chen M, Huang S. Epidemiological characteristics of dengue fever in Fuzhou, 2015. J Med Theor & Prac 2016, 29(16): 2159-62.
44. Cai F, Su C, Huang W. Surveillance of mosquitoes at Shenhu port, Jinjiang. Chinese Frontier Health Quarantine 2005, 28(3): 151-2.
45. Xu BH, Wang GD, Lin LG. Surveillance on dengue vector at Wuyishan City in Fujian Province, China. Chinese J Vector Biol Control 2003, 14(2): 127-9.
46. Huang K, Xu J-Yi, Hong C-Z, Yu Y, Lai T-R. The Surveillance report of vector of transmission of dengue fever at Zhaoyin Port District of Zhangzhou. Sci Travel Med 2006, 12(4): 25-7.
47. Wang ZH, Zheng HN. Investigation of *Aedes* mosquito density in Zhangzhou. Chinese J Vector Biol Control 2004, 15(2): 57-8.
48. Xu G, Wang Z. Investigation of dengue vectors in Zhangzhou. Strait J Prey Med 2008, 14(6): 58-59.
49. Xu R, Wang G-A, Ma X, Zhao T-Y. An investigation and analysis of *Aedes albopictus* breeding situation in Ningbo area 2011-2015. Acta Parasitol Med Entomol Sin 2016, 23(2): 105-110.
50. Shu L-P, Zuo L, Zhao X, Chen A-Y, Wei L-H. Susceptibility of 15 collections of *Aedes albopictus* from Guizhou to dengue virus oral infection. Chinese J Exp Clin Virol 2004, 18(3): 234-8.
51. Jiang Y, Song F, Li C, Li N, Du H, Li J, Fan D. Investigation on mosquitoes and study on natural infection of the *Encephalitis* B virus at Dalian Port. Chin J Frontier Health Quarantine 2009, 32(5): 363-7.
52. Li HB, Qi FJ. Distribution of *Aedes albopictus* in Dalian. Chinese J Vector Biol Control 2003, 14(1): 67.
53. Wang F, Liu G-P, Ren Q-M, Han X-N, Sun W, Liu Y. Investigation of mosquito species from three provinces of Northeast China. Chin J Vector Bio & Control 2006, 17(6): 476-80.
54. Wang X, Pan L. Study on the distribution of *Aedes albopictus* in Chaoyang City. Chin J Vector Bio & Control 2007, 18(2): 140.
55. Wang S, Chen H, Li L, He Z, Dai X, Wang X, Ye S. Report on the monitoring and control of mosquitoes at Shuangliu airport, Chengdu. Chinese Frontier Health Quarantine 2008, 31(suppl): 23-27.
56. Xu Q, Li J, Liu Z. Population density and structure of rats, mosquitoes and flies at Tianjin seaport from 1998-2000. Port Health Control 2001, 6(4): 7-11.
57. Huang J. Surveillance of dengue vector populations in Jingdezhen city, 2015. Medical Information 2016, 29(19): 206-7.
58. Wang G, Zhou K, Chen H, Cheng Z, Liu X, Wan Y, Liu F. Baseline investigation on flies and mosquitoes at Nanchang port. Chin J Frontier Health Quarantine 2001, 24(5): 287-9.
59. Chen Y. Investigations on the adult mosquito density and population dynamics in residential areas along railway in Jiayuguan. Lit & Inf Prev Med 2002, 8(6): 656-7.
60. Li G, Li H, Ren X, Liu J. Investigations on the population density of dengue vector *Aedes albopictus* along Yangtze River in Gansu province. Health Profession Education 2012, 30(2): 129-30.
61. Lie P, Wang Z, Li R, Liu K. Population density and dynamics of mosquitoes in Hanyin county of Ankang city. Vet Med Prev 2006, 22(2): 111-3.
62. Liu P, Wang Z. Mosquitoes species and population dynamics in Ankang city. Chin J Vector Bio & Control 2008, 19(6): 578-80.
63. Han X, Zhang Y, Liu Z, Li L. Distribution of *Aedes albopictus* in Shaanxi province. Chinese J Hygienic Insecticides and Equipment 2015, 4: 434-5.
64. Li X-L, Sun Y-X, Zhang B-F, Zhang Z, Liu Y, Wang S-J. A survey on distribution and density of *Aedes albopictus* in Baoji city. Chin J Vector Bio & Control 2007, 18(6): 453-5.
65. Wu H⁃X, Lu L, Meng F⁃X, Guo Y⁃H, Liu Q⁃Y. Reports on national surveillance of mosquitoes in China, 2006-2015. Chin J Vector Biol & Control 2017, 28(5): 409-15.
66. Shi Y-Y, Zhang J-M. Preliminary investigation on geographic distribution of *Aedes albopictus* in northwest China. Chinese Journal of Hygienic Insecticides & Equipments, 2012, 18(05): 420-1.
67. Wang S, Ding J, Zhang J, Wang X, Liu C, Han L. The distribution of *Aedes albopictus* in the northern of Liaoning province. Chinese J Vector Biol & Control 2009, 20(03): 191-2.
68. Sun Y-X, She J-J Study on dividing line and influence factor of *Aedes albopictus* in Shaanxi province. Chinese Journal of Vector Biology and Control 2009, 20(05): 430-2.
69. Li C, Wang L, Wu X, Liang Y, Yan L, Liu C. Preliminary report on the distribution of *Aedes albopictus* in Shenyang. Chinese Journal of Vector Biology and Control 2010, 21(03): 291.
70. Liu Y, Li X, Chen C. Monitoring and analysis on the Northern distribution of *Aedes albopictus* and investigate the density of border. Chinese J Pest Control 2009, 5: 324-7.
71. Li G, Fu H, Guo Y, Jia Y, Zhao G. The first report of dengue vector *Aedes albopictus* in the Jialingjiang river region of Gansu province. Chinese J Nat Med 2009, 11(02): 128-9.
72. Li G-T, Fu H, Guo Y-H, Shen M-X, Ren X-M, Li H-L, Ding X-Q. Geographical distribution of *Aedes albopictus* in the Yangtze River basin in Gansu province. Chinese Journal of Vector Biology and Control 2010, 21(3): 248-9.
73. Wang Z-M, Xing D, Wu Z-M, Yao W-J, Gang W, Xin D-S, Jiang Y-F, Xue R-D, Dong Y-D, Li C-X, Guo X-X, Zhang Y-M, Zhao T-Y. Biting activity and host attractancy of mosquitoes (Diptera: Culicidae) in Manzhouli, China. J Med Entomol 2012, 49(6): 1283-8.
74. Cao Y, Fu S, Tian Z, Lu Z, He Y, Wang H, Wang J, Guo W, Tao B, Liang G. Distribution of mosquitoes and mosquito-borne arboviruses in Inner Mongolia, China. Vector Borne Zoonotic Dis 2011, 11(12): 1577-81.
75. Li Y, Zhu J, Li H. Distribution of the dengue fever vector in Xishuangbanna Prefecture of Yunnan. China Trop Med 2016, 16(3): 237-65.
76. Yang MD, Jiang JY, Zheng YT, Zhou HN. Distribution survey on *Aedes aegypti* in the border areas of Yunnan province, China. Chin J Vector Biol & Control 2015, 26(4): 406-8.
