# Supplementary material for: Seasonality modeling of the distribution of Aedes albopictus in China based on climatic and environmental suitability
Source: Infect Dis Poverty. 2019 Dec 3;8:98. doi: 10.1186/s40249-019-0612-y (PMC6889612; doi:10.1186/s40249-019-0612-y)
Supplement: Supplementary file 4 — Additional file 4: Figure S1. Map of China with provinces and their boundaries. Bottom right box: South China Sea islands. [file 40249_2019_612_MOESM4_ESM.pptx]

## Slide 1
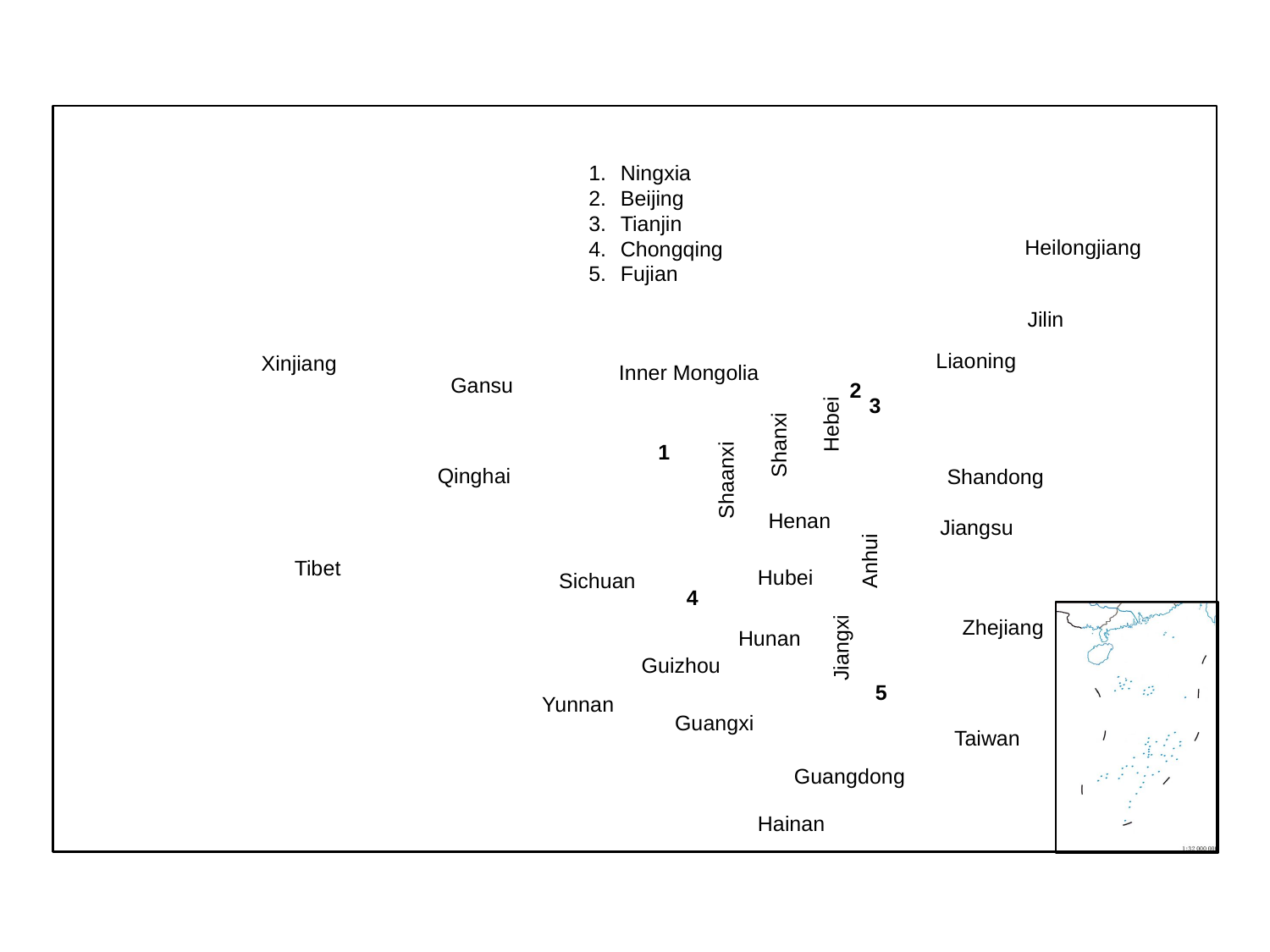

Ningxia
Beijing
Tianjin
Chongqing
Fujian
Heilongjiang
Jilin
Liaoning
Xinjiang
Inner Mongolia
Gansu
2
3
Hebei
Shanxi
1
Qinghai
Shandong
Shaanxi
Henan
Jiangsu
Anhui
Tibet
Hubei
Sichuan
4
Zhejiang
Hunan
Jiangxi
Guizhou
5
Yunnan
Guangxi
Taiwan
Guangdong
Hainan
