# Supplementary figures and images for: Seasonality modeling of the distribution of Aedes albopictus in China based on climatic and environmental suitability
Source: Infect Dis Poverty. 2019 Dec 3;8:98. doi: 10.1186/s40249-019-0612-y (PMC6889612; doi:10.1186/s40249-019-0612-y)

## Slide 1
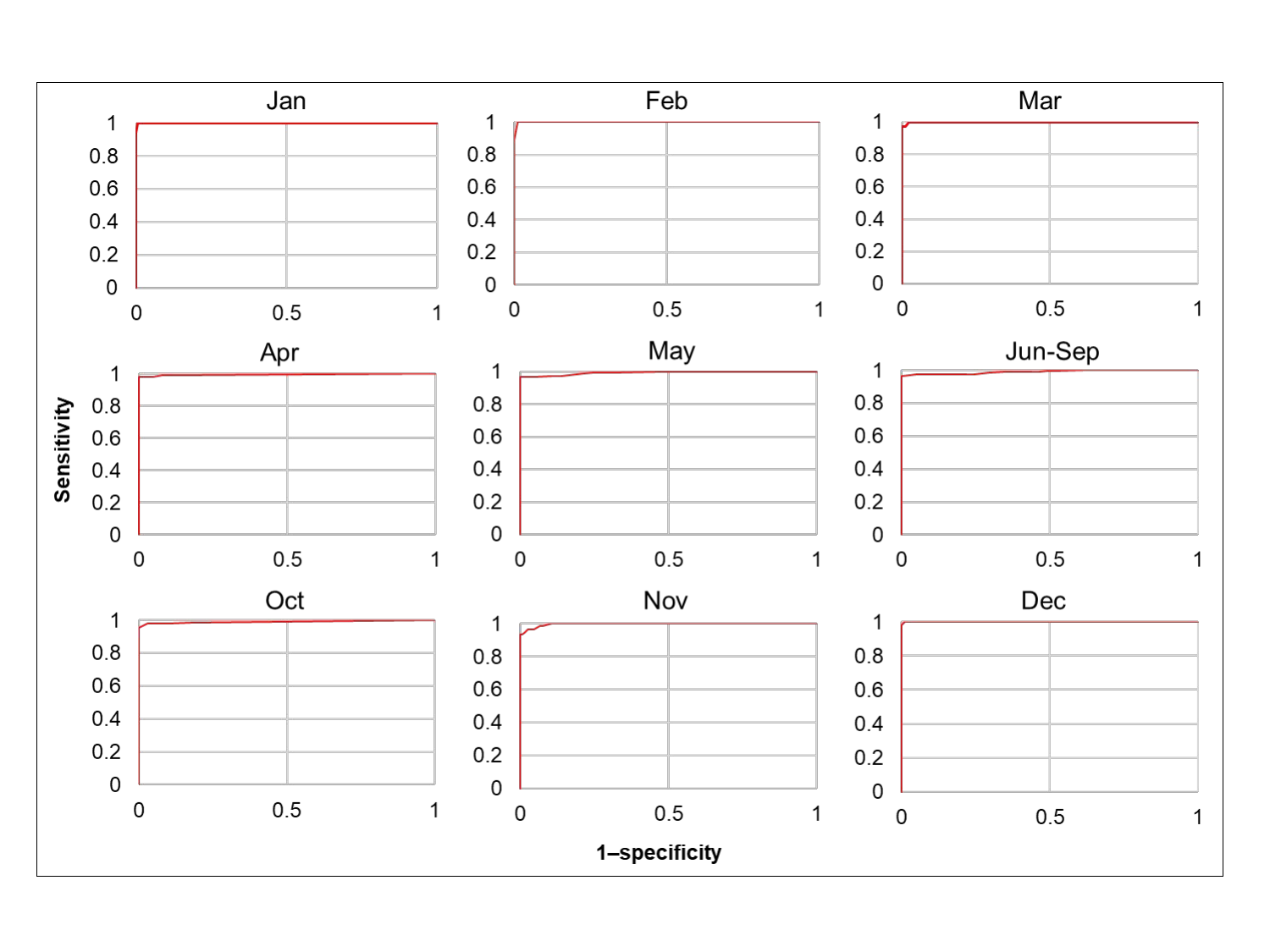

Supplement: Supplementary file 5 — Additional file 5: Figure S2. Graphs of receiver operating characteristic curves (ROCs) for each month or cluster of months. [file 40249_2019_612_MOESM5_ESM.pptx]
